# 14-3-3ζ deficient mice in the BALB/c background display behavioural and anatomical defects associated with neurodevelopmental disorders

**DOI:** 10.1038/srep12434

**Published:** 2015-07-24

**Authors:** Xiangjun Xu, Emily J. Jaehne, Zarina Greenberg, Peter McCarthy, Eiman Saleh, Clare L. Parish, Daria Camera, Julian Heng, Matilda Haas, Bernhard T. Baune, Udani Ratnayake, Maarten van den Buuse, Angel F. Lopez, Hayley S. Ramshaw, Quenten Schwarz

**Affiliations:** 1 Centre for Cancer Biology, SA Pathology and University of South Australia, Frome Road, Adelaide, 5000, Australia; 2 Discipline of Psychiatry, University of Adelaide, Adelaide, SA 5005, Australia; 3 The Florey Institute of Neuroscience and Mental Health, The University of Melbourne, Parkville, 3010, Australia; 4 School of Medical Sciences, RMIT University, Bundoora, 3083, Australia; 5 Harry Perkins Institute of Medical Research, Perth, Australia; 6 School of Medicine and Pharmacology, University of Western Australia, Crawley, 6009, Australia; 7 Australian Regenerative Medicine Institute, Monash University, Clayton, Australia; 8 School of Psychological Science, La Trobe University, Melbourne, Australia

## Abstract

Sequencing and expression analyses implicate 14-3-3ζ as a genetic risk factor for neurodevelopmental disorders such as schizophrenia and autism. In support of this notion, we recently found that 14-3-3ζ^−/−^ mice in the Sv/129 background display schizophrenia-like defects. As epistatic interactions play a significant role in disease pathogenesis we generated a new congenic strain in the BALB/c background to determine the impact of genetic interactions on the 14-3-3ζ^−/−^ phenotype. In addition to replicating defects such as aberrant mossy fibre connectivity and impaired spatial memory, our analysis of 14-3-3ζ^−/−^ BALB/c mice identified enlarged lateral ventricles, reduced synaptic density and ectopically positioned pyramidal neurons in all subfields of the hippocampus. In contrast to our previous analyses, 14-3-3ζ^−/−^ BALB/c mice lacked locomotor hyperactivity that was underscored by normal levels of the dopamine transporter (DAT) and dopamine signalling. Taken together, our results demonstrate that dysfunction of 14-3-3ζ gives rise to many of the pathological hallmarks associated with the human condition. 14-3-3ζ-deficient BALB/c mice therefore provide a novel model to address the underlying biology of structural defects affecting the hippocampus and ventricle, and cognitive defects such as hippocampal-dependent learning and memory.

Neurodevelopmental disorders arise from aberrant embryonic and postnatal brain development and encompass a wide spectrum of pathologies such as schizophrenia, autism and intellectual disability. Although heterogeneous in nature, recent sequencing analyses have shown that many of these disorders, in particular schizophrenia and autism, arise from mutations in overlapping molecular pathways thereby suggesting they share similar pathophysiological origins^1^,^2^,^3^. Indeed, this notion is further supported by the finding that these disorders often share similar anatomical features including structural anomalies of the hippocampus, enlarged ventricles and reduced synaptic density^4^,^5^,^6^,^7^.

The family of 14-3-3 proteins comprise seven distinct isoforms (β, ζ, ε, γ, η, τ, σ) that are expressed abundantly throughout development and in adult tissue^8^. This family of proteins comprise over 1% of total soluble brain protein^9^ and have been implicated in several neurological disorders such as epilepsy^10^, bipolar disorder^11^,^12^, mental retardation^13^ and lissencephaly. Notably, non-synonomous *14-3-3ζ* mutations have recently been identified in both schizophrenia and autistic patients^14^,^15^. Taken together with the findings that this gene is consistently down-regulated in post-mortem brain samples at the mRNA^11^,^16^ and protein levels^17^,^18^,^19^, these data collectively identify *14-3-3ζ* as a potential risk factor for neurodevelopmental disorders.

Mouse models of schizophrenia and autism have been paramount in our understanding of genetic risk factors and in the identification of biological pathways underlying neurobehavioural deficits. To define the role of 14-3-3ζ in neurodevelopmental disorders we recently characterised some of the anatomical, physiological and behavioural defects of 14-3-3ζ^−/−^ mice in the Sv/129 background^20^,^21^. Our findings demonstrated that 14-3-3ζ is required for normal brain development and brain function. Thus, 14-3-3ζ^−/−^ mice have schizophrenia-like behavioural defects including hyperactivity and disrupted sensorimotor gating that are accompanied by aberrant neuronal migration and axonal guidance defects in the hippocampus^20^. We further demonstrated that baseline hyperactivity of 14-3-3ζ^−/−^ mice arises from aberrant dopamine signaling as a result of decreased levels of the dopamine transporter (DAT). Given that 14-3-3ζ^−/−^ mice respond favourably to the frontline antipsychotic drug clozapine, our previous findings suggest that 14-3-3ζ^−/−^ mice represent a novel neurodevelopmental model of schizophrenia and associated disorders.

One of the major confounding factors in interpreting findings from neurodevelopmental mouse models is the epistatic effects of the background strain. For example, the phenotypes of mice lacking DAT vary dramatically depending on genetic background^22^ and even wild type mice from different backgrounds have profoundly different behavioural phenotypes^23^,^24^. To establish the role of candidate genes in the pathophysiology of any particular disorder it is therefore essential to examine the role of any mutations in multiple genetic backgrounds.

To investigate the contribution of genetic backgrounds to the 14-3-3ζ^−/−^ phenotype we have derived a new congenic strain in the BALB/c background by back-crossing to this line for over 10 generations. BALB/c mice were chosen as they are a widely used inbred mouse strain that breeds well and shows markedly different behavioural phenotypes to the Sv/129 strain^21^,^23^,^24^. Moreover, as BALB/c mice are reported to respond differently to psychostimulants acting on the dopamine pathway^23^ this line provides an ideal model to test the role of 14-3-3ζ in dopamine signalling.

Here we report that 14-3-3ζ^−/−^ mice in the BALB/c genetic background replicate all of the anatomical defects previously reported in the Sv/129 strain and uncover additional hallmark phenotypes of neurodevelopmental disorders. In the absence of profound structural brain defects BALB/c mice lacking 14-3-3ζ display mispatterning of hippocampal pyramidal neurons and misrouting of dentate mossy fibres. Importantly, we show for the first time that 14-3-3ζ is essential for correct formation of the lateral ventricles and for hippocampal synaptic connections. Consistent with physiological dysfunction of the hippocampus we found that 14-3-3ζ^−/−^ mice had striking neurobehavioural deficits in spatial learning and memory. In contrast, we failed to observe alterations in anxiety or locomotor function, a finding that is underscored by normal levels of DAT and dopaminergic signaling. Our analysis therefore shows that 14-3-3ζ is essential for neurodevelopment and for higher-order brain function. Although 14-3-3ζ^−/−^ mice have variable behavioural phenotypes depending on the genetic background, each of the lines present unique features of human neurodevelopmental disorders and identify a key role for deficiency of the 14-3-3ζ molecular pathway in the pathophysiology of schizophrenia and associated disorders.

## Results

### 14-3-3ζ-deficient mice in the BALB/c background display hippocampal defects

We have previously shown that 14-3-3ζ^−/−^ mice in the Sv/129 background display anatomical and behavioural defects reminiscent to neurodevelopmental disorders such as schizophrenia^20^,^21^. To determine if different genetic backgrounds affect the 14-3-3ζ^−/−^ phenotype we back-crossed these mice in to the BALB/c background for over 10 generations. Quantitative RT-PCR and western blot analysis on embryonic and adult tissue confirmed that BALB/c 14-3-3ζ^−/−^ mice completely lacked *14-3-3ζ* expression (Supplementary Fig. 1). Inter-crosses of BALB/c 14-3-3ζ heterozygous mice gave rise to homozygous mice at the expected Mendelian ratio at birth indicating that 14-3-3ζ^−/−^ mice are embryonically viable (25.5% 14-3-3ζ^+/+^, 53.2% 14-3-3ζ^+/−^, 21.2% 14-3-3ζ^−/−^; n = 470, P = 0.1772). Notably, back-crossing into the BALB/c background rescued the growth retardation and postnatal death of 14-3-3ζ^−/−^ mice that was evident in the Sv/129 background. Regardless of the genetic background 14-3-3ζ^−/−^ males and females were infertile. Tests of olfaction, vision, balance, self-righting, eye blink, eye twitch, whisker orientation and neuromuscular strength were normal in 14-3-3ζ^−/−^ compared to 14-3-3ζ^+/+^ controls (summarised in Table S1) further indicating that there are no outwardly abnormal phenotypes in these mice.

**Figure 1:**
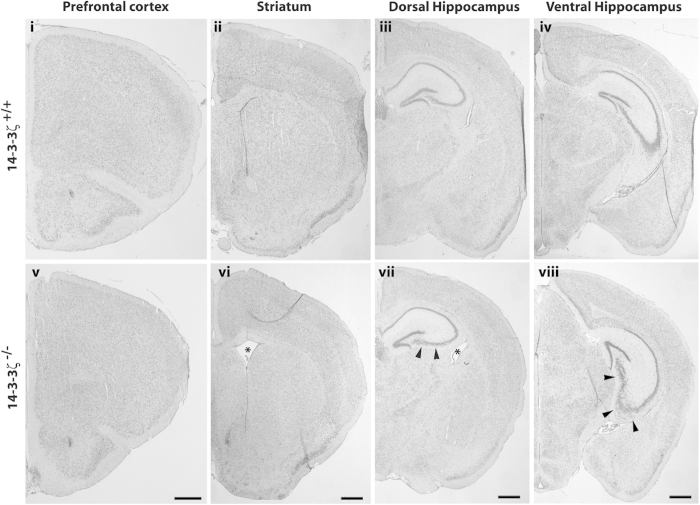
Structural brain defects in 14-3-3ζ-deficient mice. Nissl staining of coronal brain sections from 5 month old adult 14-3-3ζ^+/+^ (i–iv) and 14-3-3ζ^−/−^ mice (v–viii). Images show that lamination of the prefrontal cortex (i and v), motor cortex and cingulate cortex (i–iii and v–vii), thalamus (iii and vii) and amygdala (iii and vii) are normal in 14-3-3ζ^−/−^ mice. Lamination defects were identified in the dorsal and ventral hippocampus of 14-3-3ζ^−/−^ mice (iii and vii, iv and viii respectively; arrowhead). The lateral ventricle was also enlarged in 14-3-3ζ^−/−^ mice (ii–iii and vi–vii; asterisk). Scale bar = 100 μm.

Nissl staining of coronal brain sections from adult mice indicated that laminar organisation of several brain regions, implicated in neurodevelopmental disorders, such as the prefrontal cortex, cingulate cortex, hypothalamus, motor cortex and striatum formed normally in BALB/c 14-3-3ζ^−/−^ mice (Fig. 1). In contrast, pyramidal neurons of the dorsal and ventral hippocampus in cornu ammonis (CA) subfields CA1, CA2 and CA3 were ectopically positioned (arrowheads, Fig. 1 iii,iv,vii,viii). Consistent with the notion that 14-3-3ζ^−/−^ mice model schizophrenia-like symptoms, Nissl staining also uncovered enlargement of the lateral ventricle in 14-3-3ζ^−/−^ adult mice compared to wildtypes, that was fully penetrant and not previously identified in other genetic backgrounds (Fig. 1vi,vii and ii,iii respectively; n = 5/genotype).

**Figure 2:**
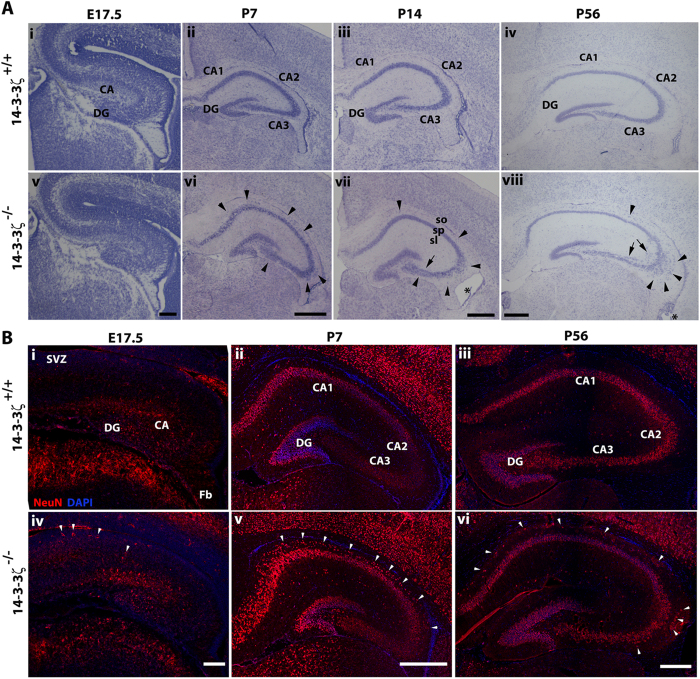
Hippocampal lamination defects in 14-3-3ζ-deficient mice. (**A**) Nissl staining shows the hippocampal development of 14-3-3ζ^+/+^ (i–iv) and 14-3-3ζ^−/−^ (v–viii) mice from embryonic day (E) 17.5 until post natal day (P) 56. During embryonic development no gross structural defects could be seen in 14-3-3ζ^−/−^ mice (**i** and **v**). Pyramidal neurons in the cornu ammonis (CA) subfields, CA1-3, were dispersed in the stratum pyramidale (sp) and stratum oriens (arrowheads) layer in 14-3-3ζ^−/−^ mice. Arrows highlight the duplicated layer of hippocampal pyramidal neurons in stratum radiatum (sr). Scale bar = 100 μm. (**B**) Coronal sections of the hippocampus obtained from E17.5 (**i** and **iv**), P7 (**ii** and **v**) and P56 (iii and vi) 14-3-3ζ^+/+^ and14-3-3ζ^−/−^ mice. At E17.5 the stratum pyramidale is populated by NeuN-positive pyramidal cells in 14-3-3ζ^+/+^ hippocampi forming a uniform mature zone in the developing CA. In 14-3-3ζ^−/−^ hippocampi, the maturation zone was less uniform with some NeuN-positive mature pyramidal cells ectopically positioned in the superficial zone of the stratum pyramidale in the CA and others in the sub ventricular zone (SVZ; white arrowheads). In P7 and P56 14-3-3-ζ^−/−^ mice, NeuN immunostaining identifies mature pyramidal neurons ectopically positioned in the stratum oriens of CA1-3 (arrowheads). Scale bars: 100 μm.

We next asked if the neuronal mispatterning and enlarged ventricles arose from developmental defects. Ectopically positioned pyramidal neurons in CA1-3 were first identified prior to hippocampal maturation at postnatal day (P) 7 and this was maintained throughout all postnatal stages (asterisk, Fig. 2A; 3/3 at P7, 5/5 at P14 and 5/5 at P56). Similar to our observations in the Sv/129 background, pyramidal neurons within the CA3 subfield split into a bilaminar stratum while pyramidal neurons within the CA2/3 boundary were ectopically positioned in the stratum radiatum and stratum oriens. In addition, we identified ectopically positioned neurons in the stratum oriens of the CA1 subfield that was not previously described in Sv/129 14-3-3ζ^−/−^ mice^20^ (Fig. 2Avi–viii). Analysis of Nissl-stained sections was unable to detect any notable differences at embryonic day (E) 17.5, a stage at which the pyramidal neurons are starting to condense in to a bonafide stratum (Fig. 2Ai and v). Enlargement of the lateral ventricles was also identified during developmental stages, first being detected at P14 with no notable defects at P7 (Fig. 2Avi–viii) or in additional mice examined at P10 (data not shown).

To determine if ectopically-positioned pyramidal cells formed mature neurons we next immunostained coronal sections with antibodies against the neuronal marker NeuN. At all ages examined the ectopically positioned cells were positive for NeuN (Fig. 2B) indicating that these neurons likely form functional connections. At E17.5 when pyramidal neurons are condensing in to the stratum of the CA in the 14-3-3ζ^+/+^ controls, mature neurons were identified in the superficial layers juxtaposing the subventricular zone of 14-3-3ζ^−/−^ embryos (Fig. 2Bi,ii). Taken together, and in light of our previous work in the Sv/129 background, these findings identify a clear role for 14-3-3ζ during hippocampal morphogenesis and pyramidal neuron migration.

### Aberrant hippocampal connectivity in 14-3-3ζ-deficient mice

**Figure 3:**
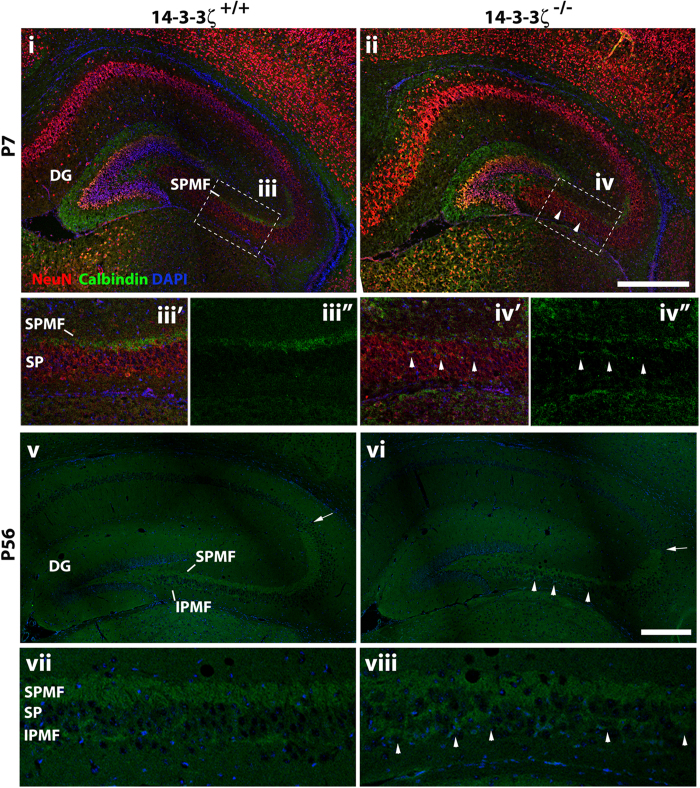
Abnormal connectivity of the hippocampus in 14-3-3ζ-deficient mice. Calbindin immunostaining of the infrapyramidal (IPMF) and the suprapyramidal (SPMF) mossy fibre trajectories. At P7 (i–iv) the calbindin mossy fibres are seen along the SPMF branch navigating away from the dentate gyrus (DG) in both 14-3-3ζ^+/+^ and 14-3-3ζ^−/−^ mice. In 14-3-3ζ^−/−^ mice the SPMF (green) branch aberrantly navigates among the NeuN positive (red) pyramidal cell somata (sp, arrowheads) in CA3. (iii’) higher magnification of boxed area in (i) and (iii”) higher magnification of calbindin staining from boxed area in (i). (iv’) Higher magnification of boxed area in (ii) and (iv”) higher magnification of calbindin staining from boxed area in (ii). At P56 (v-viii) the SPMF and IPMF branches of 14-3-3ζ^−/−^ mice navigate aberrantly among the pyramidal cell somata (arrowheads) in CA3 and, the SPMF is shorter than in 14-3-3ζ^+/+^ mice (arrow). (vii and viii) higher magnification of the boxed regions in (v) and (vi), respectively. Scale bars = 100 μm.

Functional connectivity within the hippocampus is essential for high-order brain function^25^. Given the ectopic positions of neurons within the CA of 14-3-3ζ^−/−^ mice we next asked if the mossy fibre connections between the dentate granular neurons and CA3 pyramidal neurons were also affected. Immunostaining with antibodies against calbindin showed that the suprapyramidal and infrapyramidal mossy fibre tracts were aberrantly aligned in the 14-3-3ζ^−/−^ mice, compared to wildtype littermates (Fig. 3i–iv). Whereas the suprapyramidal tract formed tight axonal bundles along the apical surface of the CA3 pyramidal neurons in 14-3-3ζ^+/+^ mice, these mossy fibres navigated within the CA3 pyramidal layer of 14-3-3ζ^−/−^ mice at P7 (Fig. 3iii,iv). In adult 14-3-3ζ^+/+^ brains, mossy fibres split into tightly bundled infrapyramidal and suprapyramidal branches lining the CA3 pyramidal layer. However, in 14-3-3ζ^−/−^ mice the suprapyramidal fibres were diffuse and the infrapyramidal branch aberrantly navigated among the pyramidal cell layer and ectopically fused with the suprapyramidal branch (Fig. 3v–viii). Moreover, analysis of calbindin staining showed that the suprapyramidal branch failed to extend to the boundary of CA2/3 in 14-3-3ζ^−/−^ adult brains (arrowheads, Fig. 3v,vi).

**Figure 4:**
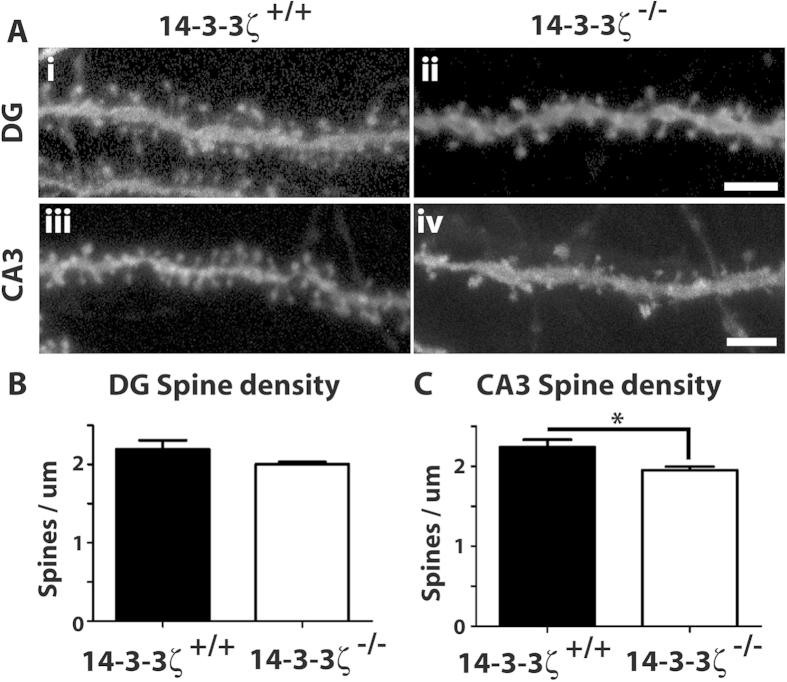
14–3–3ζ-deficient mice have reduced spine density. (**A**) Biolistic labelling of dendritic spines in the dentate gyrus (DG) and cornu ammonis layer 3 (CA3) of 14-3-3ζ^+/+^ and 14-3-3ζ^−/−^ mice. (**B**) Granular neurons in the DG have similar numbers of dendritic spines in 14-3-3ζ^−/−^ (open bar) and 14-3-3ζ^+/+^ mice (closed bar; n = 3 mice/genotype, >50 dendrites quantified/mouse). (**C**) Pyramidal neurons in CA3 have significantly reduced dendritic spine density in 14-3-3ζ^−/−^ mice (open bar) compared to 14-3-3ζ^+/+^ mice (closed bar; n = 3 mice/genotype, >50 dendrites counted/mouse). * <0.05. Scale bar = 5μm.

Given the aberrant axonal navigation within the hippocampus we next asked if the granular and pyramidal neurons had altered synaptic density. Dendritic spines are small membranous protrusions on neuronal dendrites that mark the sites of contact between pre- and post-synaptic neurons. As over 90% of excitatory synapses form on these spines their density and morphology are considered a direct correlation of synaptic strength and activity^26^,^27^. Furthermore, altered spine formation is associated with several human conditions showing deficits in social interaction, cognition and memory function, including schizophrenia, autism and intellectual disability^28^,^29^,^30^,^31^. To analyse spine density we labelled individual neurons in fixed vibratome sections with biolistic delivery of lipophilic fluorescent dyes (DiI and DiO). Spines from secondary apical dendrites on CA3 pyramidal neurons and basal dendrites from dentate granular neurons were quantitated from 3D-reconstructed confocal images. In comparison to 14-3-3ζ^+/+^ adult mice, we identified reduced spine density in 14-3-3ζ^−/−^ mice that was specific to the CA3 region of the hippocampus (Fig. 4; n = 3/genotype with over 50 dendrites counted/mouse, P = 0.04). 14-3-3ζ is therefore required for the formation of functional connections within the hippocampus.

### 14-3-3ζ-deficient mice display cognitive defects

**Figure 5:**
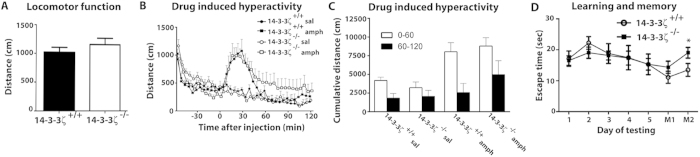
14–3–3ζ-deficient mice in the BALB/c background demonstrate abnormal cognitive traits. (**A**) 14-3-3ζ^−/−^ mice (open bars; n = 12; 5 male and 7 female) have similar exploratory behaviour at 3 months of age compared to 14-3-3ζ^+/+^ littermates (filled bars; n = 10; 5 male and 5 female) in an open field test. (**B**) 14-3-3ζ^−/−^ mice (open circle; n = 12; 5 male and 7 female) have similar baseline exploratory behaviour compared to 14-3-3ζ^+/+^ littermates (closed circle; n = 10; 5 male and 5 female) in an open field test over 120 mins. 14-3-3ζ^−/−^ mice and 14-3-3ζ^+/+^ mice display similar hyperactivity in response to amphetamine (5 mg/kg). 14-3-3ζ^−/−^ mice showed a trend towards increased hyperactivity in the 60–120 min post amphetamine injection compared to 14-3-3ζ^+/+^ mice. (**C**) 14-3-3ζ^−/−^ mice and 14-3-3ζ^+/+^ mice have no significant differences in amphetamine-induced hyperactivity in the 0–60 min or 60–120 min post amphetamine injection. Error bars are presented as mean ± SEM. (**D**) 14-3-3ζ^−/−^ mice (closed squares; n = 12; 8 male and 4 female) and 14-3-3ζ^+/+^ mice (open circles; n = 8; 4 male and 4 female) have similar capacity for spatial learning (Day1-5) in a cross maze escape task test. In contrast, 14-3-3ζ^−/−^ mice have reduced capacity to remember (M1 and M2) in the cross maze escape task.

As the anatomical defects of 14-3-3ζ^−/−^ mice are conserved across genetic backgrounds we next asked if the BALB/c model recapitulates the schizophrenia-like behavioural defects observed in the Sv/129 background. We first tested the locomotor function of 14-3-3ζ^−/−^ mice in an open field environment. In contrast to the robust hyperactivity seen in the Sv/129 background, 14-3-3ζ^−/−^ in the BALB/c background showed no differences in distance travelled over the test period (Fig. 5A), or across sexes (data not shown). We next performed an analysis of amphetamine-induced hyperactivity. Both WT 14-3-3ζ^+/+^ and 14-3-3ζ^−/−^ mice demonstrated a decline in activity in the test arena during the 60 min habituation phase. Consistent with previous reports suggesting that BALB/c mice respond poorly to psychostimulants of the dopamine signalling pathway^23^, subcutaneous injection of amphetamine (5 mg/kg) induced only mild and variable hyperactivity in both 14-3-3ζ^+/+^ (n = 10; 5 male, 5 female) and 14-3-3ζ^−/−^ mice (n = 12; 5 male, 7 female), with similar time to become maximally hyperactive and similar degree of hyperactivity being reached across both genotypes and sexes (Fig. 5B). 14-3-3ζ^−/−^ mice trended toward covering a greater distance in the 60–120 min post amphetamine injection, however the accumulated distance travelled in this period was not significantly different between genotypes (Fig. 5C).

To test the levels of anxiety, we next analysed the mice on the elevated zero maze. 14-3-3ζ^−/−^ mice and 14-3-3ζ^+/+^ mice showed similar preference for the closed quadrants over the open quadrants of the maze (Supp. Fig. 2A). As the anxiety levels of BALB/c mice are heightened under normal lighting conditions compared to other strains^32^, we also completed the elevated plus maze test under low-level lighting. Consistent with our previous test of anxiety, we found that 14-3-3ζ^−/−^ mice and 14-3-3ζ^+/+^ mice showed similar preference for the closed arm to the open arms of the maze in the 5 min test period with no change in rearing or head dipping (Supp. Fig. 2B). Therefore, in contrast to our previous findings in the Sv/129 background, mice lacking 14-3-3ζ in the BALB/c background do not display disturbances of hyperactivity or anxiety.

Given the structural defects in the hippocampus we next completed a series of tests to investigate cognitive behaviours of learning and memory. Short-term memory of 14-3-3ζ^−/−^ mice was first explored by performing an object recognition task. Overall both the 14-3-3ζ^+/+^ and 14-3-3ζ^−/−^ mice had limited interactions with the novel and familiar objects with 14-3-3ζ^−/−^ mice trending towards spending less time interacting with the objects compared to controls (Supp Fig. 3A). However, both genotypes showed the same preference for the novel object compared to the familiar object, as indicated by an equivalent preference index (Supp. Fig. 3B). We next explored working memory-dependent learning and memory using a cross-maze escape task under low-level lighting to reduce anxiety levels of the BALB/c mice. After 5 days of training to identify the correct arm of a cross-maze containing a submerged escape platform, we found that 14-3-3ζ^−/−^ mice learned at the same rate as 14-3-3ζ^+/+^ mice (Fig. 5D), showing the same amount of latency to find the escape platform. However, only the 14-3-3ζ^+/+^ mice had a significant change in escape latency during the learning phase, suggesting that they learned better than 14-3-3ζ^−/−^ mice (Fig. 5D, One-way ANOVA, P = 0.0116). The ability to remember the correct arm of the maze was then tested after a 14- and 28-day rest period (M1 and M2, respectively). Consistent with defects in hippocampal-dependent memory, we found that 14-3-3ζ^−/−^ mice had significantly increased escape latency compared to 14-3-3ζ^+/+^ mice after the 28-day rest period (Fig. 5D; student t-test, P = 0.04).

### Dopamine signalling is preserved in BALB/c mice lacking 14-3-3ζ

**Figure 6:**
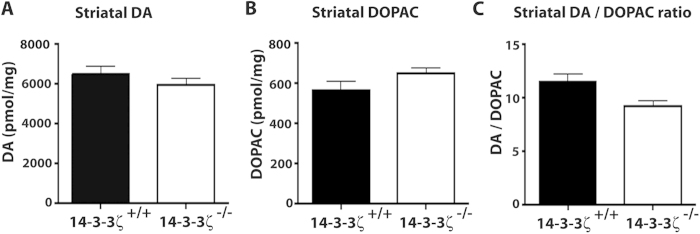
Baseline dopamine levels are conserved in 14-3-3ζ-deficient BALB/c mice. (**A**) Baseline DA and DOPAC levels, measured in the striatum by HPLC. 14-3-3ζ^−/−^ mice (open bar; n = 12; 7 male and 5 female), were not significantly different from 14-3-3ζ^+/+^ mice (closed bar; n = 10; 5 male and 5 female). (**B**) 14-3-3ζ^−/−^ mice have the same levels of DOPAC compared to 14-3-3ζ^+/+^ mice. (**C**) Dopamine turnover (DOPAC/DA ratio) was conserved in 14-3-3ζ^−/−^ mice compared to 14-3-3ζ^+/+^ mice.

We have previously shown that baseline hyperactivity of 14-3-3ζ^−/−^ mice in the Sv/129 background correlates with reduced levels of the dopamine transporter DAT and increased levels of dopamine^21^. As 14-3-3ζ^−/−^ in the BALB/c background lacked hyperactivity, we tested the notion that DAT and dopamine are also preserved in this model. Notably, both dopamine signalling and DAT function have been reported to be altered in BALB/c mice^23^,^33^. Dopamine signalling is usually balanced by reuptake from the synaptic space through DAT, which promotes recycling to the presynaptic vesicular pool or degradation to DOPAC and other by-products. Total tissue levels of dopamine and DOPAC were measured in the striatum, cortex and hypothalamus by reverse phase high performance liquid chromatography with electrochemical detection (HPLC). Our analysis found that tissue content of dopamine, DOPAC and dopamine turnover (ratio of dopamine/DOPAC) were preserved across genotypes in all regions examined (Fig. 6A–C and Supp. Fig. 4), supporting our previous behavioural analyses (Fig. 5).

**Figure 7:**
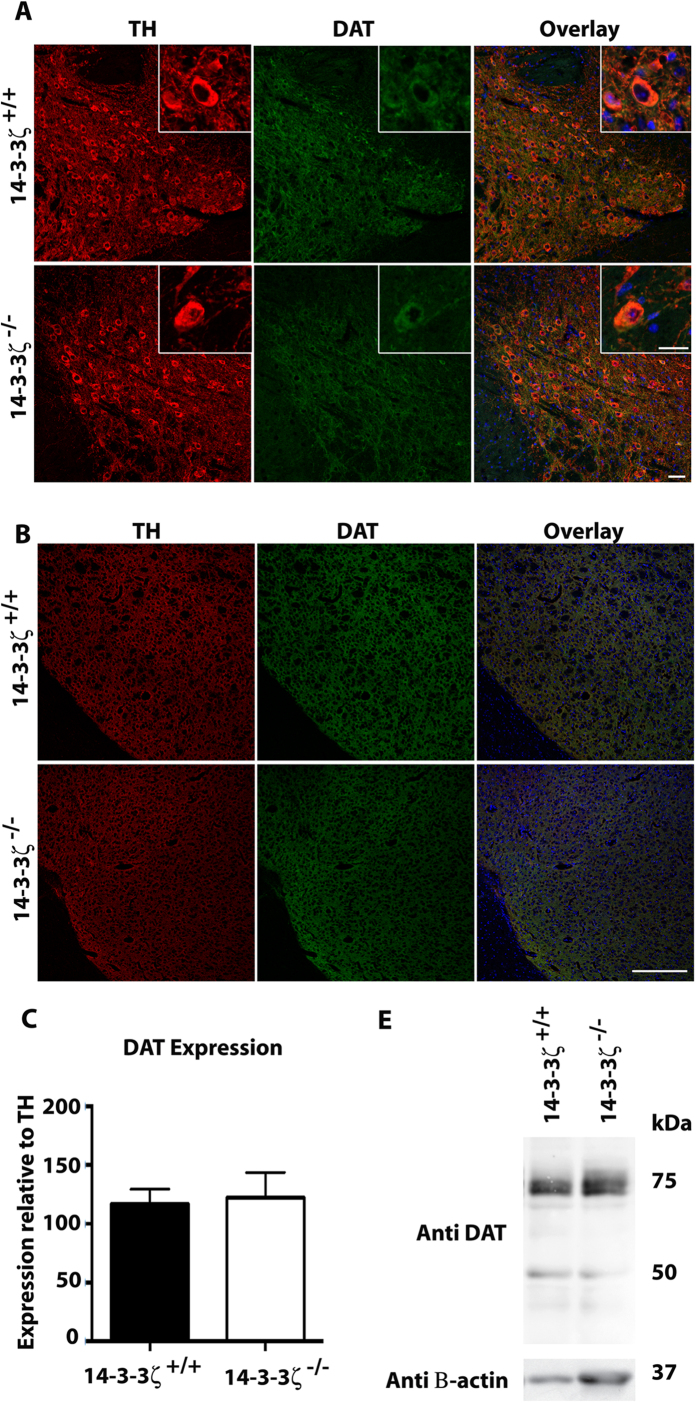
Expression of DAT is conserved in 14-3-3ζ-deficient mice in the BALB/c background. (**A**) Sagittal brain sections stained with anti-TH (red) and anti-DAT (green) show similar levels of DAT in the VTA/SN of 14-3-3ζ^−/−^ mice compared to 14-3-3ζ^+/+^ littermates. Higher magnification of anti-DAT immunostaining (inserts) in VTA/SN shows that DAT is localised normally in dopaminergic neurons. Scale bars = 50 μm in main figure and 20μm in the higher magnification inset figure. (**B**) Sagittal brain sections show similar levels of DAT in the striatum of 14-3-3ζ^−/−^ mice compared to 14-3-3ζ^+/+^ littermates. Scale bars = 200μm. (C) Quantitation of anti-DAT immunostaining in the striatum normalised to anti-TH confirms that 14-3-3ζ^−/−^ (open bar; n = 4) had equivalent expression of DAT compared to 14-3-3ζ^+/+^ mice (closed bar; n = 4). (D) Western blot analysis of whole brain lysate shows that proteins levels of unglycosylated DAT (50 kDa) and glycosylated DAT (80 kDa) are equivalent in 14-3-3ζ^−/−^ (n = 2) 14-3-3ζ^+/+^ mice (n = 2).

We next examined the localisation and abundance of DAT by co-labelling sagittal brain sections with anti-DAT and anti-TH antibodies. In contrast to our previous findings in the Sv/129 model, we observed no changes in the expression levels or localisation of DAT within the SN-VTA of 14-3-3ζ^−/−^ mice (Fig. 7A). Thus, DAT was distributed evenly throughout the cell body and neurites of dopaminergic neurons within the midbrain in both 14-3-3ζ^+/+^ and 14-3-3ζ^−/−^ mice. Expression of DAT was quantified by measuring the fluorescence intensity of anti-DAT in the termini of dopaminergic neurons within the striatum relative to that of anti-TH antibodies. Our analysis shows that DAT levels are similar in both 14-3-3ζ^+/+^ and 14-3-3ζ^−/−^ mice (Fig. 7B,C). Analysis of adult whole-brain lysates by immunoblotting further confirmed that DAT levels were preserved in BALB/c mice lacking 14-3-3ζ (Fig. 7D).

## Discussion

Neurodevelopmental disorders such as schizophrenia and autism are highly prevalent clinical syndromes affecting over 2% of the population^34^. Although these disorders are known to arise from neurodevelopmental insults, their complex genetic nature has provided a major obstacle in elucidating the molecular and cellular deficiencies underlying their aetiology. Indeed, schizophrenia and autism are considered prototypic complex genetic traits with pathogenesis arising from synergistic defects in multiple genes within connected molecular pathways. As such, the genetic background of any affected individual plays a significant role in the pathophysiology, fecundity and severity of disease presentation. Through the use of deep sequencing technologies, several recent publications have highlighted the presence of overlapping genetic mutations in both schizophrenia and autism^1^, suggesting that many neurodevelopmental disorders share common molecular origins. Of note, exome sequencing of afflicted patients and their unaffected parents has recently identified *de novo* loss of function mutations in *14-3-3ζ* in both schizophrenia and autism^14^,^15^, strongly implicating this gene as a risk factor for neurodevelopmental disorders. Our previous analysis of 14-3-3ζ-deficient mice in the Sv/129 background provided strong support to this notion, showing that complete abrogation of 14-3-3ζ expression gives rise to schizophrenia-like behavioural, physiological and anatomical defects^20^,^21^. Importantly, our current study now shows that many of these defects are conserved across other genetic backgrounds, thereby demonstrating that these traits arise as a direct consequence of 14-3-3ζ deficiency.

In addition to replicating the hippocampal structural defects, mossy fibre navigation defects and cognitive defects previously seen in the Sv/129 background, our current study identified critical roles for 14-3-3ζ in promoting formation of the lateral ventricle and excitatory synapses. 14-3-3 regulatory proteins lack enzymatic activity but instead exert their functions by binding to substrate proteins that are phosphorylated on serine/threonine residues to modify their localisation, stabilisation and/or biological property. Our finding that pyramidal neurons of the hippocampus are ectopically positioned in 14-3-3ζ^−/−^ mice suggests that the role of 14-3-3ζ in promoting pyramidal neuron migration is conserved across genetic backgrounds. Moreover, the interactions of 14-3-3ζ with DISC1 and Ndel1^20^,^35^ further implicate this protein as a key component of the migratory machinery within this cell type. BALB/c 14-3-3ζ^−/−^ mice also had reduced dendritic spine density demonstrating that the number of excitatory synapses is reduced in these mice. Consistent with 14-3-3ζ playing an essential role in synapse formation, transgenic mice over-expressing 14-3-3ζ have been shown to have increased spine density^36^. Although it is currently unknown how 14-3-3ζ regulates spine density, it binds to several proteins found in the post synaptic density including HOMER, DISC1 and SPIN90^20^,^37^,^38^, and plays a critical role in modulating actin polymerisation by binding to cofilin^39^. Notably, mice lacking cofilin or other 14-3-3ζ interacting partners such as DAT, Ndel1 and Lis1 have reduced dendritic spine density reminiscent of what is reported here^40^,^41^.

Retrospective analysis of sections from 14-3-3ζ^−/−^ mice in the Sv/129 background confirmed that the enlarged lateral ventricle is specific to the BALB/c background (data not shown), indicating that there is a genetic modifier in one of these backgrounds that either ameliorates or enhances this defect. Alternatively, as the brain size of BALB/c mice has been reported to be larger in comparison to other backgrounds^42^, the enlarged ventricles in the 14-3-3ζ^−/−^ mice may be exacerbated in this background. Ventricular enlargement is a common occurrence in many neurodevelopmental disorders and thought to arise from aberrant cell death or reduced neurogenesis^43^. Although we can not rule out aberrant cell death as the mechanism driving ventricular enlargement, its foundations during a time at which the brain is rapidly expanding fits best with a primary defect in neurogenesis, possibly through interactions with 14-3-3ε and δ-catenin^44^.

The learning and memory defects previously identified in the Sv/129 background are also conserved in the BALB/c model^20^ and are consistent with the anatomical and synaptic defects identified in the hippocampus, a major brain centre essential for these functions. However, in contrast to these cognitive defects we did not observe altered locomotor function in the BALB/c model. Our previous analyses demonstrated that hyperactivity in 14-3-3ζ^−/−^ mice arose from aberrant DAT biogenesis and increased dopamine levels^21^. BALB/c mice have known differences in the dopamine signalling pathway that may arise from reduced expression of the monoamine oxidase enzymes MAO-A and MAO-B that also play a role in dopamine turnover^45^. Due to reduced activity of monamine oxidases, the tissue content of dopamine is increased in the BALB/c background, a defect that is thought to underpin some of the increased anxiolytic behavioural defects of these mice^46^. In addition, BALB/c mice respond poorly to psychostimulants such as amphetamine^23^. Given amphetamines primary mode of action is to block dopamine reuptake by DAT and subsequently promote dopamine efflux, there also appears to be fundamental differences in either the activity or biogenesis of DAT in the BALB/c background. In our current study we found that dopamine levels, DAT and locomotor function were normal in mice lacking 14-3-3ζ. Notably, in comparison to our previous study we found that BALB/c mice have a dramatic increase in total tissue content of dopamine (i.e. Sv/129 mice had approximately 125 pmol/mg tissue^21^ compared with BALB/c mice that had approximately 6200 pmol/mg tissue) that may also mask any effects of 14-3-3ζ in regulating the dopamine pathway. By showing that DAT levels are unaltered in the current model, our results further suggest that the genetic background of BALB/c mice is somehow protective against the loss of DAT expression observed in other 14-3-3ζ-deficient backgrounds, such as 14-3-3ζ^−/−^ Sv/129 mice. Thus, the mechanisms by which 14-3-3ζ plays a role in DAT biogenesis may be genetically heterogeneous, or there may be potentially greater redundancy for 14-3-3ζ function with respect to DAT biogenesis in BALB/c mice.

Taken together our data provide further support to the notion that deficiencies of the synapse are a key insult in the pathogenesis of behavioural deficits associated with neurodevelopmental disorders. Importantly, our analyses of 14-3-3ζ-deficiency in diverse genetic backgrounds, now demonstrates that 14-3-3ζ is essential for brain development and higher order brain function. Absence of the 14-3-3ζ molecular pathway during critical times of neuronal development is therefore predicted to underlie at least some of the anatomical and neurobehavioral deficits associated with neurodevelopmental disorders such as schizophrenia.

## Materials and Methods

### Mice

14-3-3ζ^Gt(OST062)Lex^ (or 14-3-3ζ^−/−^) mice carrying a gene trap construct that contains the βGeo reporter gene disrupting 14-3-3ζ expression have been described previously^20^. In this study we back-crossed 14-3-3ζ KO mice onto the BALB/c background for over 10 generations. *14-3-3ζ* genotype was determined by PCR amplification of genomic tail DNA as described^20^. All experiments were approved by and conducted in accordance with the guidelines of the Animal Ethics Committee of the Central Adelaide Local Health Network, the University of Adelaide and Florey Institute of Neuroscience and Mental Health.

### General health and basic sensorimotor characteristics

General physical attributes and basic sensorimotor characteristics of all animals were evaluated as previously described^47^,^48^. In brief, at the time body weight was measured, the appearance of fur and body posture were also examined. Vision was tested by placing mice on a visual cliff apparatus and by testing the ability of mice to stretch their arms to the surface upon being lowered from a 30 cm height. Olfaction was tested by the ability to locate buried cat food pellets in a clean cage in a period of 2 minutes. Balance was tested by the ability to remain standing in a shaking cage. Self-righting was tested by the ability to immediately return to a standing position after being placed on their back. Eye blink, ear twitch and whisker orientation were tested by touching the eyeball, ear or whiskers with a cotton swab. The wire-hang test was completed by placing mice on a wire cage lid and measuring the latency to fall after being inverted and held 15 cm above fresh bedding (maximum time of 60 sec).

### Behavioural assays

Except where indicated, all procedures were carried out under normal light conditions (60–100 Lux) between 8.00 am and 12.00 pm. Behavioural phenotyping was performed on the 14-3-3ζ^−/−^ and 14-3-3ζ^+/+^ BALB mice as previously described^49^,^50^,^51^. One cohort of mice was used for locomotor hyperactivity testing and measurements of dopamine/DOPAC levels at 3 months of age (14-3-3ζ^+/+^: n = 5 male and n = 5 female; 14-3-3ζ^−/−^: n = 5 males and n = 7 females). Separate cohorts of mice were used at the age of 35 weeks for the novel-object recognition test and elevated zero maze (14-3-3ζ^+/+^: n = 9 male and n = 6 female; 14-3-3ζ^−/−^: n = 7 males and n = 6 females) and at 28 weeks for the elevated plus maze and cross escape water maze (14-3-3ζ^+/+^: n = 4 male and n = 4 female; 14-3-3ζ^−/−^: n = 8 males and n = 4 females).

### Psychotropic drug-induced test of locomotor activity

Baseline locomotor activity and psychotropic drug-induced locomotor hyperactivity were assessed using an automated photobeam system (Med Associates, St. Albans, VT, USA). The system consisted of a mouse enclosure (25.4 × 25.4 × 40.6 cm) surrounded by a sensor-ring that included a 16 × 16 array of photobeams, and a computerized data-acquisition system. After 60 min of baseline locomotor activity and habituation to the test environment, amphetamine (5 mg/kg) or saline vehicle was injected intraperitoneally and behavioural activity monitored over a subsequent 120 min period.

### Novel-object recognition test

The novel-object recognition test was completed in the same apparatus as the open-field test as previously reported^52^,^53^,^54^. In brief, mice were first habituated to the apparatus for three sessions of 5 minutes each (open field test). The following day, mice were placed back into the arena for a period of 3 minutes, for the training session with 2 identical objects. Objects were black painted wooden cubes or spheres, approximately 4 cm in diameter. Following the training session (15 min), mice were placed back into the arena, for the retention session with 1 familiar object (the same as used in the training session) and 1 novel object (different shape). Time spent interacting with the 2 objects (defined as mice touching the object, or sniffing the object within a distance of 2 cm) was recorded. Total time spent interacting with objects was used as a measure of exploratory behaviour, and time spent interacting with the novel object compared to the familiar object was used as a measure of retention memory. A preference index, a ratio of the amount of time spent exploring the novel object over the total time spent exploring both objects, was used to measure recognition memory. A preference index approaching 1 was regarded as successful learning and retention memory.

### Escape water maze test

Spatial learning and memory was assessed using a cross-maze escape task as previously described^49^. The cross maze was made of clear plastic (length, 72 cm; arm dimensions, length 26 cm x width 20 cm) and placed in a circular pool of water (1 m diameter) maintained at 23°C. Milk powder was mixed with water-soluble black paint in the water to conceal a submerged (0.5 cm below the water surface) escape platform placed in the distal north arm of the maze. The pool was enclosed by a black plastic wall (height, 90 cm). Constant spatial cues were arranged at each arm of the maze and the experimenter always stood at the southern end of the apparatus during the training and testing procedures. Mice were individually habituated to the maze environment by being placed into the pool without the escape platform and allowed to swim for 60 s 2 days prior to learning. Learning trials were then conducted over a 5-day training period in which mice were required to learn the position of the submerged escape platform from the other three (East, South, West) arms that did not contain an escape platform. Each mouse was given six daily trials (two blocks of three trials separated by a 30 min rest interval), in which each of the three arms was chosen as a starting point in a randomized pattern (twice daily). For each trial, the mouse was placed in the distal end of an arm facing the wall and allowed 60 s to reach the escape platform where it remained for 10 s. Mice that did not climb onto the escape platform in the given time were placed on the platform for 10 s. The mouse was then placed in a clean holding cage before subsequent trials. Mice were assessed on their long-term retention of the escape platform location, which was placed in the same position as during the learning phase. Memory was tested 14 (M1) and 28 (M2) days after the final day of learning and consisted of a single day of 6 trials as described for the learning period. Data were recorded for each mouse for each trial on their escape latency (i.e. time (s) taken to swim to the platform), number of correct trials (i.e. if a mouse found the platform on the first arm entry) and number of incorrect entries/reentries (i.e. the number of times that a mouse went into an arm that did not contain the escape platform).

### Elevated Zero Maze

The elevated zero maze consisted of an elevated circular platform, 50 cm diameter, with a 5 cm wide platform 40 cm above ground. The zero was divided into four quadrants with two quadrants of the maze open and two enclosed with 15 cm high walls. Mice were placed into an open quadrant of the maze and were allowed to explore the apparatus for 5 minutes^55^. Time spent in the open quadrants was measured as an indication of anxiety-like behaviour.

### Elevated plus maze test

The elevated plus-maze was completed as previously described^56^,^57^. Briefly, the apparatus was made in the shape of a cross from black plexiglass and consisted of two open arms (25 cm x 5 cm) and two closed arms (25 cm x 5 cm x 16 cm) that crossed in the middle perpendicular to each other. The plus maze was raised 1 m from the ground. Individual mice were introduced to the centre of the apparatus, facing the open arm opposite to the experimenter, and were observed by video recording for 5 minutes. The number of entries into the open and closed arms, and the time in exploring both types of arm were scored. Naturalistic behaviour of the mouse, such as the number of head dipping, number of rearing and number of stretch-attended postures were also counted. After each trial, the maze was thoroughly cleaned with alcohol to remove any scents cues.

### Histology and Immunohistochemistry

For all anatomical analyses, postnatal mice were perfuse fixed with fresh 4% paraformaldehyde (PFA) dissolved in PBS as previously described^58^. Brains were rapidly dissected free from other tissue and post fixed in 4% PFA for an additional 24 hrs at 4°C. Tissue was cryopreserved in 20% sucrose at room temperature (RT) overnight and frozen in Tissue-Tek O.C.T. (Sakura Finetek, Torrance, CA). Sections were cut at a thickness of 10μm on a CM1850 cryostat (Leica) and air-dried for 60 min before staining.

Nissl staining and determination of β-galactosidase activity was performed using previously described methods^59^,^60^. For immunohistochemistry, sections were blocked in 10% non-immune goat serum or 1% bovine serum albumin in PBST (0.1 M PBS, 0.3% Triton X-100, 1% BSA) for 1 h at RT and subsequently incubated with primary antibodies for 1 h at RT. Primary antibodies and dilutions: rabbit polyclonal to calbindin-D28K (1:1000, Chemicon), mouse monoclonal to NeuN (1:500, Chemicon), rabbit polyclonal to TH (1:200; Millipore), rat monoclonal to DAT (1:20, Santa Cruz). Sections were washed several times with PBST and then incubated with 1:200 dilution of Alexa Fluor labelled secondary antibodies (Molecular Probes) or streptavidin labelled secondary antibodies (Jackson Laboratories) for 1 h at RT. After 3 washes in PBST, fluorescent sections were mounted in Prolong® Gold antifade reagent with DAPI (Molecular Probes).

### Image analysis

Low resolution images were recorded on an SZX10 stereo microscope (Olympus) equipped with a Micropublisher 3.3 digital camera (Q-Imaging) and processed with OpenLab 2.2 software (Improvision). High resolution images were captured on a LSM700 confocal microscope (Zeiss). All figures were constructed in Adobe Photoshop CS3 (Adobe Systems, Inc.). Quantification of DAT and TH expression from confocal immunofluoresecence images was completed as described previously^61^. Briefly, images were split into separate channels for TH or DAT, converted to binary images and used for fluorescence intensity calculations with an Image J area calculator macro designed to detect staining in confocal image slices.

### Immunoblotting

Nitrocellulose membranes were blocked with 2% skim milk powder in PBST and immunoblotted with Rat anti-DAT (1:500, Santa Cruz) and Mouse anti-14-3-3ζ (1:1000, Santa Cruz) (Ramshaw *et al.*, 2013). Rabbit polyclonal against β-actin (1:5000, Millipore) was used as a loading control. Bound antibodies were detected with HRP-conjugated secondary antibody (1:5,000, Pierce-Thermo Scientific). Immunoreactive proteins were visualized by ECL (Luminescent Image Analyzer LAS-4000, Fujifilm, Japan). The images were analysed with Multi Gauge Ver3.0 (Fujifilm, Japan).

### Detection of Dopamine

Dopamine and DOPAC levels in the striatum, hypothalamus and cortex were determined using HPLC as previously described^62^. For tissue preparation, small biopsies were dissected out on a chilled plate, weighed, and placed in 200 μl 0.4 M perchloric acid (HClO_4_) containing 0.05% sodium metabisulphate (Na_2_S_2_O_5_) and 0.01% disodium EDTA. The sample tissue was homogenized, cellular and vesicular membranes disrupted using a sonicator and finally stored at 70 °C. On the day of analysis, all samples were centrifuged at 10,500 *g* for 10 minutes and filtered though minispin filters for an additional 3 minutes at 10,000 rpm. The resultant supernatant was transferred to HPLC vials and placed in an autosampler for injection onto the HPLC. The HPLC consisted of a LC-20AT pump (Shimadzu), SIL-20A Autosampler (Shimadzu) and C18 reverse phase column (Bio-Rad, Hercules, USA). Detection was via a 3 mm VT-03 flow cell with glassy carbon working electrode (Antec Leyden) and Decade II Electrochemical Detector (Antec Leyden). The mobile phase consisted of 17% v/v methanol in purified deionized water containing 70 mM KH2PO4 (Merck), 0.5 mM EDTA (Merck) and 8.0 mM sulfonic acid (Merck), pH 3.0 and was run at a flow rate of 0.5 ml/min.

### Biolistic gene gun labelling and quantitation of dendritic spines

Perfused brain samples were sliced at 200 μm with a Leica VT1200 vibratome (Leica) and shot with 30 μm gold particles labelled with lipophilic tracers DiI and DiO as previously described^63^. Images were acquired at 100X magnification on an LSM 700 Zeiss confocal microscope (Zeiss) using z-stack settings overlapping at least 10% between images. Spines were counted from over 50 3D reconstructed images from between 100–500 um from the cell body from each mouse using Neuron Studio and confirmed by manual counting^64^. Spines were counted from both the CA3 subfield and dentate gyrus from 3 adult mice/genotype. The CA3 subfield and dentate gyrus regions were identified through gross morphology after staining with DAPI.

### Quantitative RT-PCR

Total RNA was isolated from total brains using Trizol (Ambion) and single stranded cDNA synthesised using the QuantiTect Reverse transcription kit (Qiagen). qPCR was performed with SYBR Green reagent (Qiagen) using the Rotor-Gene 6000 real-time PCR system (Corbett Life Science). Primers used were: *GAPDH* F: ACCCAGAAGACTGTGGATGG, R: CAGTGAGCTTCCCGTTCA; *14-3-3ζ* F: AACTGTATGGTGCCCTTCTGTGG, R: CATTCGTAGTTGTTGTTGCCCCG. Relative mRNA levels were quantified using the comparative quantitation method in Rotor-Gene 6000 Series Software. Relative mRNA levels were normalised to *GAPDH*. Each PCR was performed in technical triplicates, and each experiment was performed in at least three biological replicates for each genotype.

### Statistical analysis

All data were presented as mean ± SEM. Behavioural experiments were analysed using two-way analysis of variance (ANOVA) with repeated measures where appropriate (Systat, version 9.0, SPSS software; SPSS Inc., USA). Neurochemical data and expression analyses were analysed using ANOVA and Student’s t-test. In all studies a *p* value of <0.05 was considered to be statistically significant.

## Additional Information

**How to cite this article**: Xu, X. *et al.* 14-3-3ζ deficient mice in the BALB/c background display behavioural and anatomical defects associated with neurodevelopmental disorders. *Sci. Rep.* **5**, 12434; doi: 10.1038/srep12434 (2015).

## Supplementary Material

Supplementary Information
