# Supplementary material for: 14-3-3ζ deficient mice in the BALB/c background display behavioural and anatomical defects associated with neurodevelopmental disorders
Source: Sci Rep. 2015 Jul 24;5:12434. doi: 10.1038/srep12434 (PMC4513550; doi:10.1038/srep12434)
Supplement: Supplementary Information [file srep12434-s1.pdf]

Supplementary information

14-3-3ζ deficient mice in the BALB/c background display behavioural and anatomical defects associated with neurodevelopmental disorders

Xiangjun Xu[1], Emily J. Jaehne[2], Zarina Greenberg[1], Peter McCarthy[1], Eiman Saleh[1], Clare L. Parish[3], Daria Camera[4], Julian Heng[5,6], Matilda Haas[7], Bernhard T. Baune[2], Udani Ratnayake[3], Maarten van den Buuse[3,7], Angel F. Lopez[1], Hayley S. Ramshaw[1,*] & Quenten Schwarz[1,9,*].

[1] Centre for Cancer Biology, SA Pathology and University of South Australia, Frome Road, Adelaide, 5000, Australia

[2] Discipline of Psychiatry, University of Adelaide, Adelaide, SA 5005, Australia

[3] The Florey Institute of Neuroscience and Mental Health, The University of Melbourne, Parkville, 3010, Australia

[3]School of Medical Sciences, RMIT University, Bundoora, 3083, Australia

[5] Harry Perkins Institute of Medical Research, Perth, Australia

[6]School of Medicine and Pharmacology, University of Western Australia, Crawley, 6009, Australia

[7] Australian Regenerative Medicine Institute, Monash University, Clayton, Australia

[8] School of Psychological Science, La Trobe University, Melbourne, Australia

[9] Author for correspondence; email: quenten.schwarz@health.sa.gov.au

* equal contribution

Supplementary Table 1

Sensorimotor ability of 14-3-3$\zeta^{+/+}$ and 14-3-3$\zeta^{-/-}$ mice.

Observation	14-3-3$\zeta^{+/+}$	14-3-3$\zeta^{-/-}$
**Sensory ability**		
Visual depth	Normal	Normal
Visual cliff	Normal	Normal
Olfaction	Normal	Normal
**Neurological reflexes**		
Balance and self righting	Normal	Normal
Eye blink and ear twitch	Normal	Normal
Whisker-orienting	Normal	Normal
**Neuromuscular strength**		
Wire-hang test	60 sec	57.7 sec

The occurrence of sensorimotor abilities is defined as "normal". Wire hang tests are represented as mean with a maximum end point of 60 sec allowed for the test. (14-3-3$\zeta^{+/+}$, n = 6; 14-3-3$\zeta^{-/-}$, n = 11).

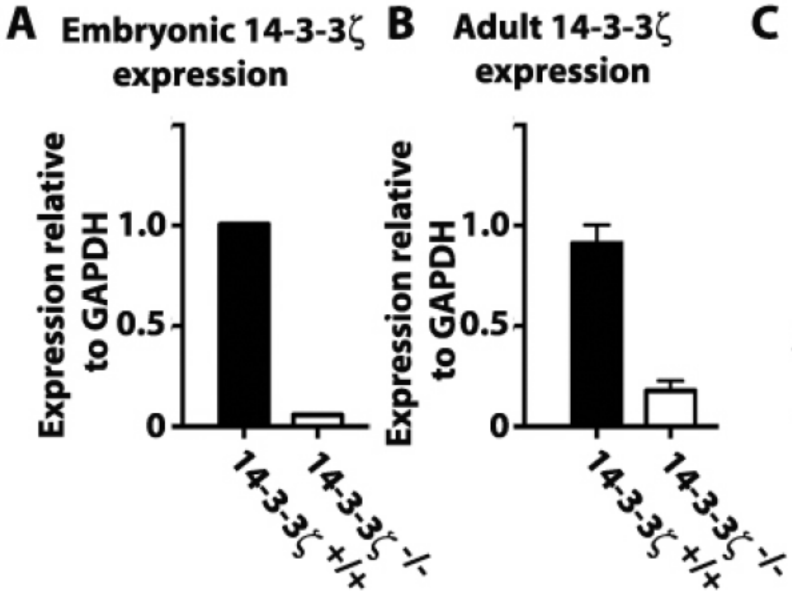

Supplementary Figure 1. Expression analysis of 14-3-3ζ in 14-3-3ζ$^{-/-}$ BALB/c mice.

(A-B) Quantitative RT-PCR of *14-3-3ζ* in 14-3-3ζ$^{+/+}$ and 14-3-3ζ$^{-/-}$ E15.5 (A) and adult (B) brain tissue (n=3/genotype). (C) Western blot analysis of 14-3-3ζ from 14-3-3ζ$^{+/+}$ and 14-3-3ζ$^{-/-}$ adult brain tissue (n=6/genotype). Data represents Mean <u>+/-</u> SEM.

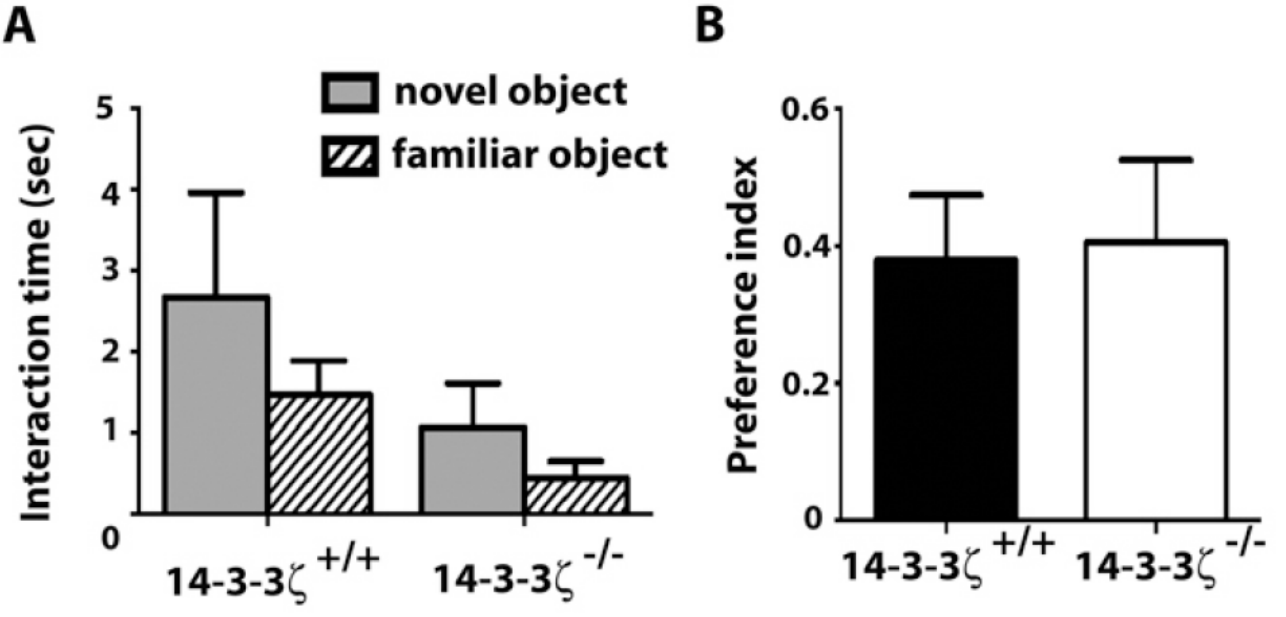

Supplementary Figure 2. 14-3-3ζ-deficient mice in the BALB/c background perform normally in the novel object recognition task.

(A) 14-3-3$\zeta^{-/-}$ mice (n=13; 7 male and 6 female) have reduced overall interaction time with the novel and familiar objects in the novel object recognition task but have similar preference for the novel object compared to 14-3-3$\zeta^{+/+}$ littermates (n=15; 9 male and 6 female). (B) Preference for the novel object is the same between 14-3-3$\zeta^{-/-}$ mice and 14-3-3$\zeta^{+/+}$ littermates.

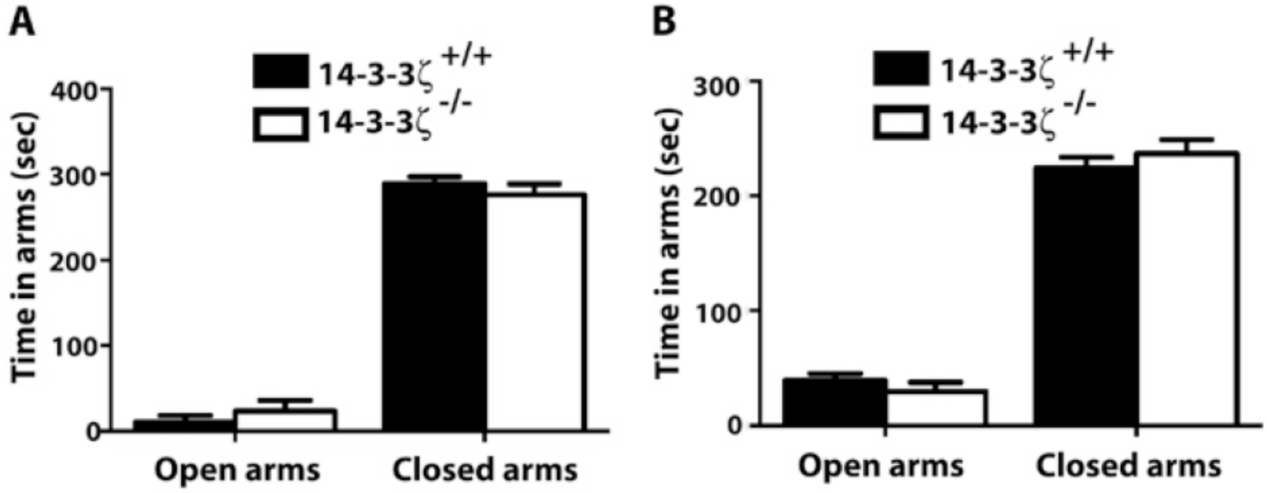

Supplementary Figure 3. 14-3-3ζ-deficient mice in the BALB/c background demonstrate normal levels of anxiety.

(A) Compared to 14-3-3$\zeta^{+/+}$ mice (filled bars; n=8; 4 male and 4 female), 14-3-3$\zeta^{-/-}$ mice (open bars; n=8; 4 male and 4 female) spend the same time in the open and closed arms of an elevated plus maze. (B) Compared to 14-3-3$\zeta^{+/+}$ mice (filled bars; n=15; 9 male and 6 female), 14-3-3$\zeta^{-/-}$ mice (open bars; n=13; 7 male and 6 female) spend the same time in the open and closed arms of an elevated zero maze.

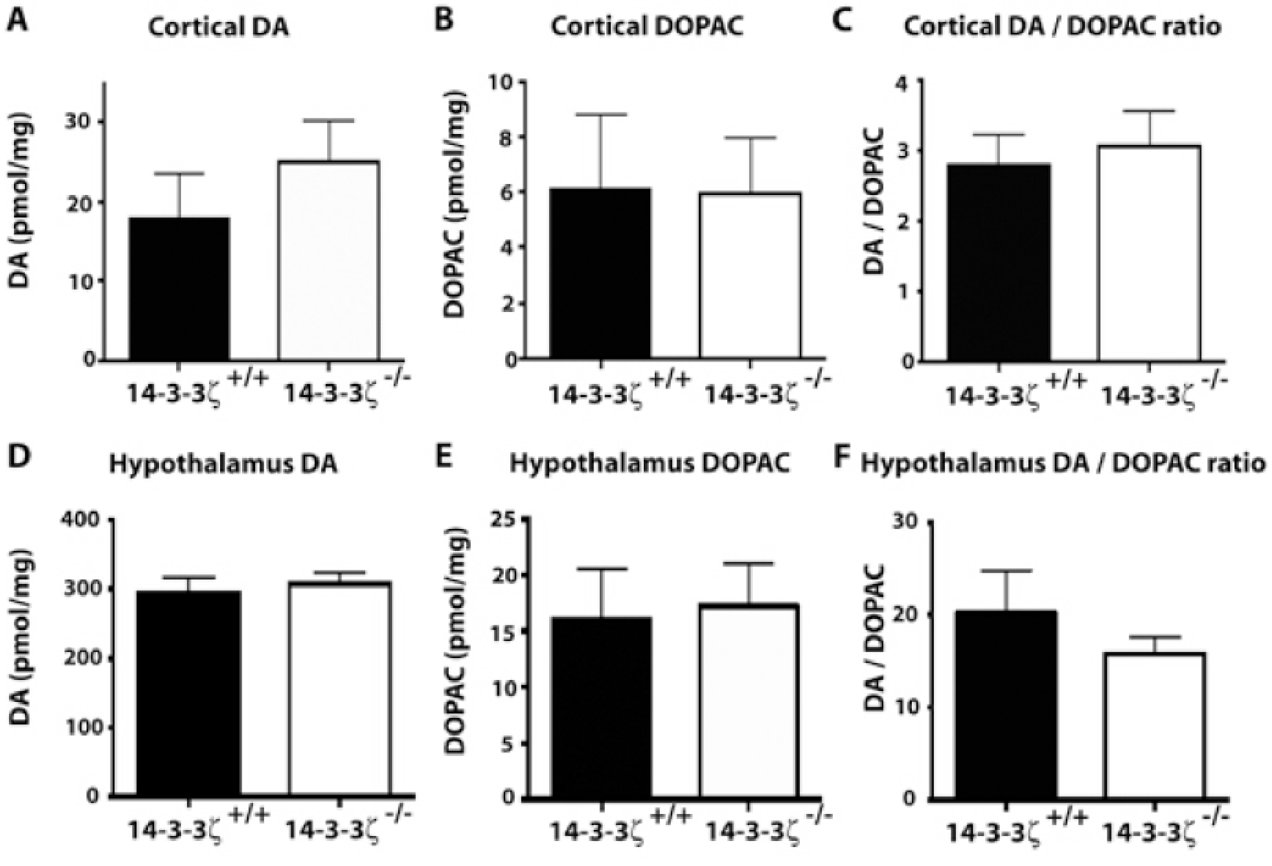

Supplementary Figure 4. Baseline levels of dopamine and DOPAC in 14-3-3ζ-deficient mice in the BALB/c background. Baseline DA and DOPAC levels were measured in the cortex (A-C) and hypothalamus (D-F) by HPLC. (A) 14-3-3$\zeta^{-/-}$ mice (open bar; n=12; 7 male and 5 female) have similar levels of DA compared to 14-3-3$\zeta^{+/+}$ mice (closed bar; n=10; 5 male and 5 female) in the cortex. (B) DOPAC levels are similar between 14-3-3$\zeta^{-/-}$ mice (open bar) and 14-3-3$\zeta^{+/+}$ mice (closed bar) in the cortex. (C) Dopamine turnover (DOPAC / DA ratio) was similar between 14-3-3$\zeta^{-/-}$ mice (open bar) compared to 14-3-3$\zeta^{+/+}$ mice (closed bar) in the cortex. (D) 14-3-3$\zeta^{-/-}$ mice (open bar) had similar levels of DA compared to 14-3-3$\zeta^{+/+}$ mice (closed bar) in the hypothalamus. (E) DOPAC levels were similar between 14-3-3$\zeta^{-/-}$ mice (open bar) and 14-3-3$\zeta^{+/+}$ mice (closed bar) in the hypothalamus. (F) Dopamine turnover (DOPAC / DA ratio) is similar between 14-3-3$\zeta^{-/-}$ mice (open bar) compared to 14-3-3$\zeta^{+/+}$ mice (closed bar) in the hypothalamus.
